# Platypus predation has differential effects on aquatic invertebrates in contrasting stream and lake ecosystems

**DOI:** 10.1038/s41598-020-69957-1

**Published:** 2020-08-03

**Authors:** Tanya A. McLachlan-Troup, Stewart C. Nicol, Christopher R. Dickman

**Affiliations:** 1 School of Life and Environmental Sciences, University of Sydney, Sydney, NSW 2006 Australia; 2 School of Natural Sciences, University of Tasmania, Hobart, TAS 7001 Australia

**Keywords:** Ecosystem ecology, Freshwater ecology

## Abstract

Predators can have strong impacts on prey populations, with cascading effects on lower trophic levels. Although such effects are well known in aquatic ecosystems, few studies have explored the influence of predatory aquatic mammals, or whether the same predator has similar effects in contrasting systems. We investigated the effects of platypus (Monotremata: *Ornithorhynchus anatinus*) on its benthic invertebrate prey, and tested predictions that this voracious forager would more strongly affect invertebrates—and indirectly, epilithic algae—in a mesotrophic lake than in a dynamic stream ecosystem. Hypotheses were tested using novel manipulative experiments involving platypus-exclusion cages. Platypuses had strongly suppressive effects on invertebrate prey populations, especially detritivores and omnivores, but weaker or inconsistent effects on invertebrate taxon richness and composition. Contrary to expectation, predation effects were stronger in the stream than the lake; no effects were found on algae in either ecosystem due to weak effects of platypuses on herbivorous invertebrates. Platypuses did not cause redistribution of sediment via their foraging activities. Platypuses can clearly have both strong and subtle effects on aquatic food webs that may vary widely between ecosystems and locations, but further research is needed to replicate our experiments and understand the contextual drivers of this variation.

## Introduction

Predation can strongly influence the size and dynamics of prey populations and the structure of prey communities^1–3^. Such influences can arise if predators selectively depredate prey species from the array of prey taxa that is available (consumptive effects,e.g. Garvey, et al.^4^, or if predator-presence influences the behaviour or other traits of prey individuals (non-consumptive effects,e.g. Preisser, et al.^5^. These effects of predators were first noted in limnological studies in the 1960s^6,7^, and subsequent research has confirmed predation to be a major structuring force within many freshwater communities^8–10^. Some invertebrates^11^, as well as many species of fish and other vertebrates, have strong predatory effects^12,13^. Indeed, Morin^14^ argued that vertebrate predation was the cornerstone of aquatic community theory, and an overview of aquatic and terrestrial ecosystems found that the strongest interactions occurred in association with invertebrate herbivores and endothermic vertebrate predators^15^.

The effects of predators often extend beyond their immediate prey to lower trophic levels. Such 'trophic cascades' have been defined as “indirect species interactions that originate with predators and spread downward through food webs”^16^. For example, a predator that directly suppresses populations of herbivorous prey species could be expected to indirectly favour plant biomass and diversity^17,18^. It has been argued that such predator effects are greater^19^ and act over shorter timescales^16^ in aquatic than terrestrial systems, and are greater in lentic benthos than in streams^19^. Several mechanisms have been proposed to explain the observed difference in strength of trophic cascades between lentic and lotic food webs, and these are based largely on attributes of the predator and prey species and the systems that they occupy. Thus, ecosystems with trophic cascades are usually characterised by discrete and homogeneous habitats, rapid prey population dynamics compared to predator dynamics, and simple trophic stratification^20^. These conditions can facilitate strong interactions between species and increase local biodiversity^20^. Environmental homogeneity should also favour keystone-type predation (sensu Paine^21^, with streams considered to be more heterogenous than lakes. Low biodiversity also can be important in driving trophic cascades^22^: mesotrophic lakes and low biodiversity ecosystems are most likely to demonstrate cascading effects between trophic levels^23^. Cascading effects are also produced if interaction strengths are large, and target species monopolise the available resources^21^.

A recent review^24^ has stressed the importance of cross-taxonomic and cross-system approaches to provide broader views of the role of predators in ecosystems, hence, studies on the same top predator species in contrasting ecosystems should be of considerable value. We take this approach here, and examine specifically the influence of a mammalian top predator— the platypus (Monotremata: *Ornithorhynchus anatinus*)—on the structure of invertebrate communities in lotic and lentic systems in eastern Australia.

Platypuses are medium-sized (body mass: males 0.8–3 kg, females 0.6–1.7 kg^26^, semi-aquatic, largely nocturnal predators that feed almost exclusively on benthic invertebrates^27–30^. Platypuses may forage continuously for over half the diel cycle^31^ with up to 75 foraging dives per hour^32^, consuming 13–19% of their body weight each day^33,34^. During late lactation daily food intake can reach 90–100% of body weight^35^. Although distributed widely in freshwater systems in eastern Australia, platypus numbers are declining, and the species' status is not secure^25^.

Given their high metabolic requirements and, in some locations, high abundance, platypuses are likely to have strong impacts on benthic invertebrate communities through direct consumption and by eliciting predator avoidance behaviours. As platypuses may select for different invertebrates^29^, they could potentially alter invertebrate community composition such as by reducing the abundance of herbivores which may, in turn, trigger trophic cascades that extend to autotrophs^36,37^. To investigate these possibilities, we explored whether the presence of platypuses influenced both invertebrates and epilithic algae in a westerly flowing stream in New South Wales (lotic) and a sub-alpine lake in Tasmania (lentic). In addition, platypus foraging behaviour, which involves vigorous overturning of substrata by the forepaws and sifting by the bill, disturbs aquatic substrata and sediments^38^. As this activity may move and redistribute sediments, it could further affect the microhabitats used by benthic invertebrates^39^, this possibility was also examined.

Using the platypus as a model predator in these contrasting lentic and lotic ecosystems, our specific hypotheses were that platypuses would:

1. Reduce the abundance and taxon richness of their invertebrate prey,
2. Have differential effects on invertebrate trophic groups and taxon composition,
3. Increase the biomass of epilithic algae by depleting herbivorous invertebrates, and
4. Move and redistribute sediments.

We also predicted that:

5. These effects of platypus would be stronger in the lentic than the lotic system.

Due to the difficulties of observing platypuses in situ, we investigated the effects of predation and associated foraging impacts using replicated exclosure-type experiments. No predation exclosure experiments have been conducted on the platypus or any other freshwater or semi-aquatic mammals, although the approach has been used on several species of terrestrial mammals^40–43^. Our comparative study using the platypus thus provides an original test of predation impacts in contrasting aquatic ecosystems and, contingent upon support for hypothesis 3, trophic cascade theory as well.

## Results

### Hypotheses 1 and 2: effects of platypuses on benthic invertebrates

Sixty-five invertebrate taxa from 17 orders were identified to genus or higher in the stream system, and 35 taxa from 12 orders in the lake. Three replicate cages were lost in the lake experiment, one from the + PLATYPUS control and two from the − PLATYPUS treatment due to water level fluctuations and possible interference by anglers.

**Figure 1:**
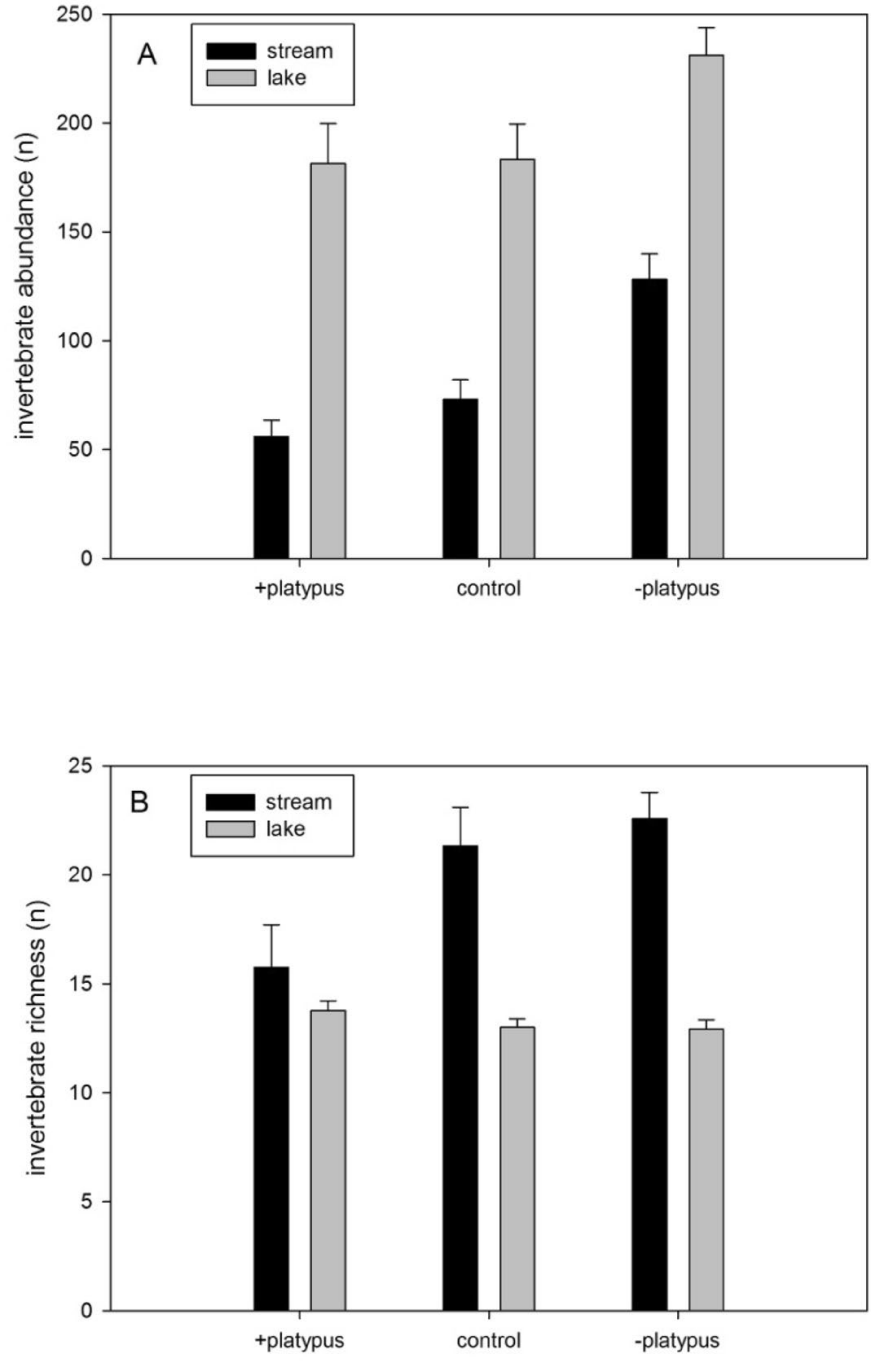
Effect of platypus activity on abundance (**A**) and taxon richness (**B**) of invertebrates in contrasting stream and lake ecosystem exclosure experiments. Stream: *n* = 36 replicates nested within three treatments; Lake: *n* = 45 replicates nested within three treatments; abundance is number of individuals per sample. Means are shown ± standard error.

In the stream system, platypus foraging had a marked effect on overall invertebrate abundance (Fig. 1A) and taxon richness (Fig. 1B) (Table 1). Invertebrate abundance was more than double in the − PLATYPUS treatment than in the + PLATYPUS treatment, while taxon richness in the + PLATYPUS treatment was 43.4% less than in the − PLATYPUS treatment; the − PLATYPUS mean also was 5.7% more than the + PLATYPUS procedural control. Tukey’s tests on invertebrate abundance confirmed that the − PLATYPUS treatment differed from both other treatments, which did not differ from each other, whereas tests on taxon richness revealed a difference only between means for the + PLATYPUS and − PLATYPUS treatments. In the lake system, by contrast, there was no effect of platypus foraging on invertebrate abundance (Fig. 1A) or taxon richness (Fig. 1B) (Table 1).

**Table 1:** Impact of platypuses on various demographic measures of stream invertebrates in exclosure experiments in contrasting stream and lake ecosystems.

System	Invertebrate demographic measure	*n*	Source	SS	df	MS	*F*	*P*
Stream	Abundance	36	Treatment	99.057	2	49.529	12.109	0.002
			Replicate within Treatment	36.806	9	4.090	1.237	0.319
			Residual	79.321	24	3.305		
Lake	Abundance	45	Treatment	30.916	2	15.458	1.724	0.220
			Replicate within Treatment	107.560	12	8.963	2.137	0.045
			Residual	125.803	30	4.193		
Stream	Taxon richness	36	Treatment	4.853	2	2.625	4.310	0.048
			Replicate within Treatment	5.477	9	0.609	1.446	0.224
			Residual	10.106	24	0.421		
Lake	Taxon richness	45	Treatment	0.129	2	0.064	0.969	0.407
			Replicate within Treatment	0.795	12	0.066	0.150	0.999
			Residual	1.333	30	0.440		
Stream	Detritivores	36	Treatment	56.295	2	28.148	18.313	< 0.001
			Replicate within Treatment	13.836	9	1.537	0.756	0.656
			Residual	48.798	24	2.033		
Lake	Detritivores	45	Treatment	2.590	2	1.295	0.324	0.729
			Replicate within Treatment	47.909	12	3.992	2.363	0.028
			Residual	50.663	30	1.689		
Stream	Herbivores	36	Treatment	8.155	2	4.078	2.923	0.105
			Replicate within Treatment	12.553	9	1.395	2.637	0.028
			Residual	12.707	24	0.529		
Lake	Herbivores	45	Treatment	1.906	2	0.953	1.515	0.259
			Replicate within Treatment	7.543	12	0.629	0.837	0.613
			Residual	22.536	30	0.751		
Stream	Omnivores	36	Treatment	15.882	2	7.941	9.386	0.006
			Replicate within Treatment	7.520	9	0.846	0.851	0.579
			Residual	23.851	24	0.994		
Lake	Omnivores	45	Treatment	6.647	2	3.323	0.309	0.739
			Replicate within Treatment	128.668	12	10.722	3.270	0.004
			Residual	98.347	30	3.278		
Stream	Predators	36	Treatment	2.044	2	1.022	0.903	0.439
			Replicate within Treatment	10.175	9	1.131	2.166	0.063
			Residual	12.518	24	0.522		
Lake	Predators	45	Treatment	35.657	2	17.828	2.517	0.122
			Replicate within Treatment	84.996	12	7.083	13.942	< 0.001
			Residual	15.252	30	0.508		

Data √-transformed, nested 3-treatment ANOVA.

**Figure 2:**
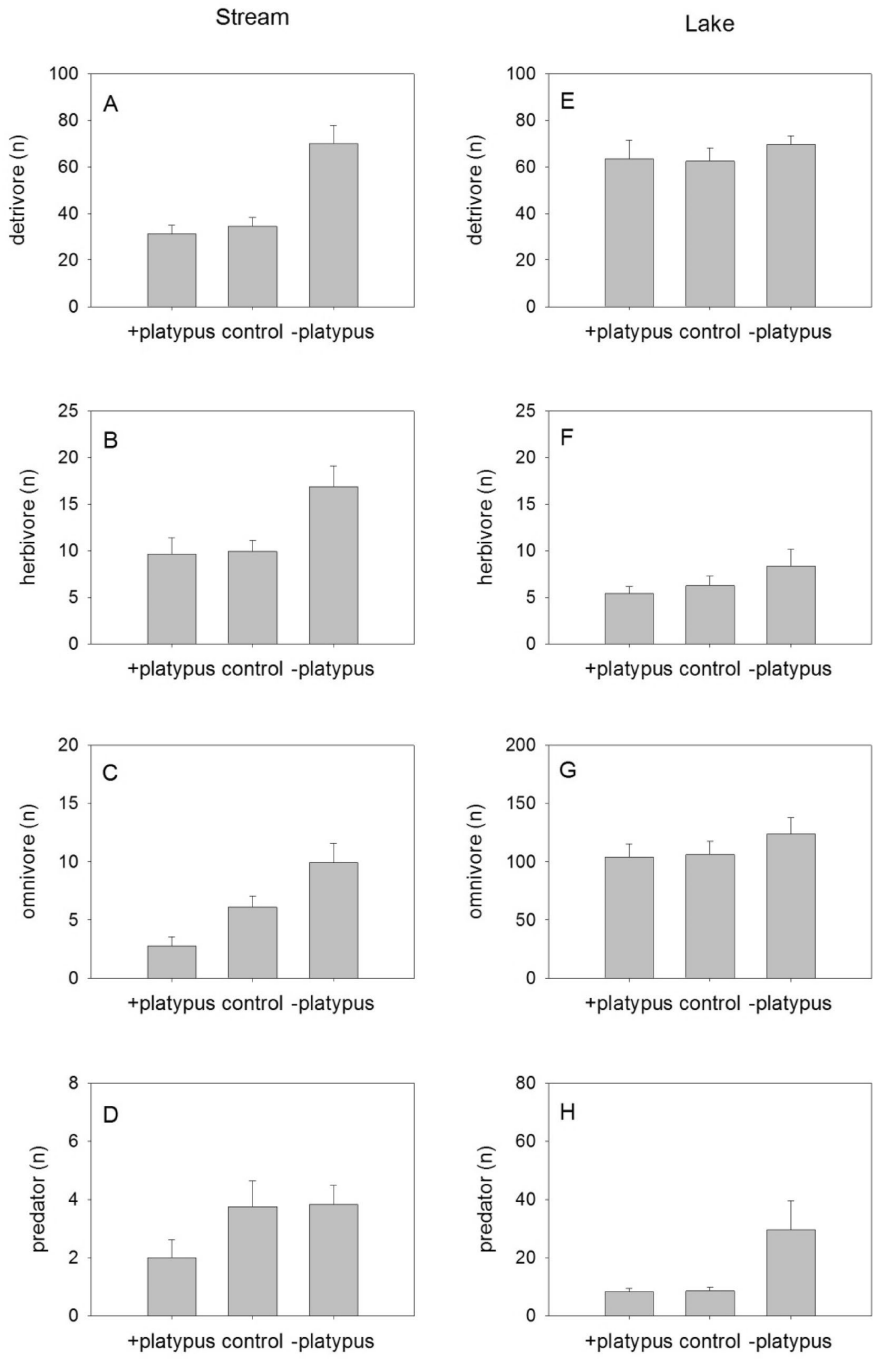
Effect of platypuses on abundance of invertebrate trophic groups in two contrasting exclosure experiments. (**A**–**D**) stream; (**E**–**H**) lake; (**A**,**E**) detritivores; (**B**,**F**) herbivores; (**C**,**G**) omnivores (note different scales); (**D**,**H**) predators (note different scales). Stream: *n* = 36 replicates, nested within 3 treatments. Lake: *n* = 45 replicates, nested within three treatments. Means are shown ± standard error.

Similar patterns were observed in trophic groups of invertebrates (Fig. 2). In the stream, detritivore abundance was over 100% higher in the absence of platypuses than in either the + PLATYPUS or + PLATYPUS control treatments (Table 1, Fig. 2A). In the lake system, by contrast, there was no effect of platypus foraging on detritivorous invertebrates, although a significant nesting effect in the lake experiment suggested variation among replicate cages nested within treatments (Table 1, Fig. 2E). Invertebrate herbivore abundance was not affected by the presence of platypuses in either the stream (Table 1, Fig. 2B) or the lake systems (Table 1, Fig. 2F). The presence of platypuses had a strongly negative effect on omnivorous invertebrates within the stream system (Table 1, Fig. 2C), but no impact on this trophic group in Lake Lea (Fig. 2G). There was, however, a significant effect of replicate nested within treatment in the latter system (Table 1). In the stream experiment the presence of platypuses had no effect on invertebrate predators (Table 1), although the overall abundance of this trophic group was low relative to the other trophic groups in the samples (Fig. 2D). Platypuses similarly had no effect in the lake system on the abundance of invertebrate predators (Table 1, Fig. 2H); a significant effect of replicate nested within treatment again suggested contagion in the distribution of invertebrates on the lake bed.

**Figure 3:**
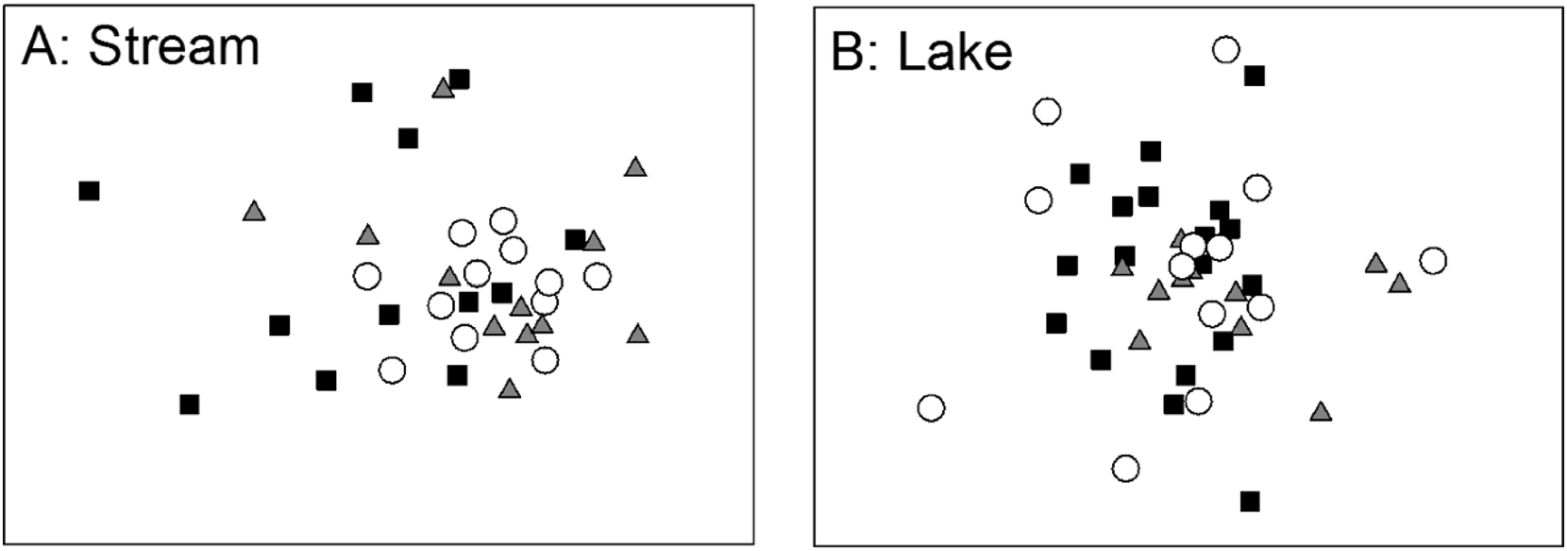
NMDS ordinations of invertebrate community data from exclosure experiments examining the impact of platypuses on benthic invertebrates. Each symbol represents one of three subsamples taken from one of four replicate cages or open benthos in the stream experiment, or from the six replicate cages and open benthos samples taken in the lake experiment. (**A**) Stream ordination, Stress = 0.2; (**B**) lake ordination, Stress = 0.19. (open circle = − platypus, filled rectangle =  + platypus, filled triangle = control).

No significant differences were found by ANOSIMs in the overall composition of invertebrate communities in either of the study systems, or in pairwise comparisons between the + PLATYPUS and + PLATYPUS control treatments (Table 2). However, there was a tendency (*P* = 0.06) for community composition to differ between the + PLATYPUS and − PLATYPUS treatments in the stream system, and a difference between these treatments in the lake system (Table 2). In the stream, the nMDS ordination shows the invertebrate communities to overlap broadly in composition; the − PLATYPUS group differs most, showing a narrower, less variable, spread among replicates than that shown by the other treatments (Fig. 3A). The nMDS ordination for the lake system (Fig. 3B) shows similar overlap among samples from the three experimental treatments.

**Table 2:** Overall ANOSIM of multivariate invertebrate community structure data from platypus exclosure experiments in contrasting stream and lake systems (stream: *n* = 36, Global *R* = 0.069, *P* = 0.18; lake: *n* = 45, Global *R* = 0.04, *P* = 0.096).

Comparison	Significance value Stream	Significance value Lake
+ PLATYPUS *VS* + PLATYPUS control	0.14	0.111
+ PLATYPUS *VS* − PLATYPUS	0.06	0.01

SIMPER analysis showed that the invertebrate taxa most affected by platypuses in the stream were detritivores, contributing between 5.85% and 5.13% to the observed dissimilarity between the + PLATYPUS and − PLATYPUS treatment samples (Table 3). The taxa most affected were all mayflies (Ephemeroptera): *Koornunga* sp., *Bungona* sp., and *Atalophlebia* sp. Others were Oligochaeta (worms), Chironomidae (Diptera) and *Triplectides* sp., a genus of case-building caddisflies (Trichoptera). Within the lake, the trophic groups most affected by platypuses were more diverse and represented by omnivores, detritivores, and predators; omnivorous Chironomidae and *Notalina* sp. (a case-building caddisfly from the family Leptoceridae) contributed most to the observed dissimilarity (Table 3).

**Table 3:** Results of **SIMPER** analyses of invertebrate taxa, with trophic group assignation, that contributed most to the calculated dissimilarity between + PLATYPUS and − PLATYPUS treatments in both the stream and the lake system.

System	Taxon	Trophic Group	Dissimilarity
Stream	*Koornunga* sp.	Detritivore	5.85
	Chironomidae	Detritivore	5.82
	*Bungona* sp.	Detritivore	5.58
	*Triplectides* sp.	Detritivore	5.57
	Oligochaeta	Detritivore	5.44
	*Atalophlebia* sp.	Detritivore	5.13
Lake	Chironomidae	Omnivore	13.85
	*Notalina* sp	Omnivore	10.2
	*Neoniphargus* sp.	Detritivore	8.29
	Oligochaeta	Detritivore	6.57
	*Leptoperla* sp.	Detritivore	5.96
	*Acarina* sp. 1	Predator	5.17

### Hypothesis 3: effects of platypuses on epilithic algal biomass

Platypuses had no significant cascading effect on epilithic algal biomass in either the stream or lake systems (stream: *F*_2,9_ = 0.410, *P* = 0.676, *n* = 12, √- transformed; lake: *F*_2,12_ = 0.076, *P* = 0.928, *n* = 15, √-transformed).

### Hypothesis 4: effects of platypuses on sediments

A single replicate was lost from each stream treatment due to tipping of the sediment traps. Platypus foraging, as demonstrated by the exclosure experiment in the stream, had no effect on sediment deposition (*F*_2,6_ = 1.167, *P* = 0.373, *n* = 9, √-transformed). Similarly, there was no effect of platypus activity on sediment deposition in the exclosure experiment in the lake (*F*_2,12_ = 0.094, *P* = 0.418, *n* = 15, √ transformed).

### Hypothesis 5: effects of platypuses in the lentic and the lotic system

Although formal comparisons were not made, the results show that platypuses influenced invertebrate abundance and taxon richness in the lotic system but not in the lentic system.

## Discussion

The results provide some support for our first two hypotheses, and none for the last three. The presence of platypuses had a dramatic effect on lotic invertebrate abundance, with numbers of invertebrates in the stream plots where platypuses were excluded doubling compared with those in the control plots. Invertebrate taxon richness was also greatly reduced in the presence of platypuses in the stream plots, but similarity between the + PLATYPUS control and − PLATYPUS treatments suggests that much of this difference arose due to the effects of the exclusion cages. Among trophic groups, detritivores and omnivores were most strongly depressed in the presence of platypuses, with weaker effects on herbivores. By contrast, the presence of platypuses had little evident effect on invertebrate abundance, taxon richness or trophic group assignation in the lake, even with the increased level of replication. With no support adduced for our third hypothesis, there was no detectable effect of platypus presence on epilithic algal biomass, and no effect either on sediment deposition, in both ecosystems.

Platypuses in Brogers Creek consume invertebrates from ~ 75% of the invertebrate families that are potentially available, with all trophic groups represented in the diet^29^. Hence, the suppression of detritivore and omnivore numbers by platypuses in the stream may not reflect selective predation by platypus, but rather that these trophic groups are particularly susceptible to platypus foraging. Detritivores and omnivores may lack effective predator detection or escape behaviours, although this seems unlikely to apply broadly at the trophic group level. Instead, it seems more plausible that many species in these trophic groups live in the substrata where platypus foraging is most focused^29^, thus placing them at most risk of predation. The lack of response by herbivores and predators to platypus predation may indicate, conversely, that members of these trophic groups occupy refuges under large rocks or attached to aquatic vegetation where platypuses do not gain access. Although bioturbation by vertebrates can strongly influence assemblage structure^39,56^, we found no indication that it contributed to the results here.

Detritivores and omnivores were amongst the most abundant invertebrates in both study systems, and their suppression by platypuses, at least in the stream, suggests the potential for further indirect effects. For example, fewer detritivores may mean that leaf litter and debris are broken down relatively slowly, increasing the amount of material and hence structural complexity in the stream that could be used by other organisms. Fewer detritivores may also result in a depleted prey base for other predators, such as fish and amphibians; if fewer larval forms of detritivores such as mayflies, caddis flies and chironomids translate into fewer adults, food depletion resulting from platypus foraging may extend to terrestrial predators such as birds. Although we can only speculate about such effects here, complex interactions within food webs have been described in many aquatic or terrestrial systems^1–4,8^ and, more occasionally, across the aquatic-terrestrial interface^14^. The platypus-driven interactions documented here do not indicate the existence of a trophic cascade, but they do indicate a two-trophic level predator–prey dynamic that may involve many other non-prey species via suites of indirect interactions. Uncovering such interactions would be a fruitful line of inquiry for future research, especially as platypus populations continue to decline^25^.

Despite the positive numerical response of detritivores and omnivores to platypus exclusion, the marked exclusion-cage effect on invertebrate taxon richness indicates that benthic faunal diversity responded more to the increased structural complexity that was provided by the cages than to platypus foraging. The cage effect is not wholly unexpected, as species richness is correlated with habitat complexity in many environments^57,58^. These results, as well as the small compositional difference between the + PLATYPUS and − PLATYPUS treatments, indicate that platypus foraging generally depleted taxa in the detritivore and omnivore groups without targeting particular species or genera. In addition, the richness of aquatic invertebrate species has been shown to recover faster than abundance post disturbance^59^, and this also may explain the results obtained here.

The lake system results do not preclude the possibility of selective predation, but do not provide clear evidence for selectivity either. In previous dietary studies in Lake Lea, Bethge^48^ showed that Trichoptera make up a major part of the diet of platypuses, and we also found that members of this family contributed to dissimilarity in samples between the + PLATYPUS and − PLATYPUS treatments. However, the lack of treatment effects on the abundance of any invertebrate trophic group and the contribution of three of four of these groups to compositional differences between the + PLATYPUS and − PLATYPUS treatments suggest that platypuses simply ate what they encountered during foraging.

As our experimental approach is novel in studies of semi-aquatic mammals, it is worth asking whether it yielded reliable results. The exclusion cages were designed to allow free movement of fish into and out of the cages, and to exclude only the larger platypus. However, it is possible that Australian bass in the stream or brown trout in the lake contributed to the observed impacts of platypuses on invertebrate prey if their activity in the − PLATYPUS treatments was reduced. This is unlikely for several reasons. Fish are ectothermic and so have much lower metabolic demands and, at least in the stream where the strongest impacts were detected, the numbers of platypus known to have moved through the stream study pool exceeded those of Australian bass. Moreover, both Australian bass and brown trout are primarily pelagic feeders or feed on allochthonous invertebrate sources^60,61^ and so are much less likely to affect benthic invertebrates compared with the almost exclusively benthic-feeding platypus. It is likely, therefore, that fish contributed slightly, if at all, to our results, leaving platypuses as the sole or primary predators of benthic invertebrates in this study.

Our fifth hypothesis, that platypuses would have stronger predatory—and potentially cascading—effects in the lentic than the lotic system, was based on both ecological theory and empirical observations made in other systems, yet received no support here. We briefly review past work before considering our ostensibly contradictory results.

Trophic cascades are predicted to be stronger in homogeneous habitats where there is simple trophic stratification and few species with strong interactions that occur between them^20,23^, as often found in lakes, in comparison with heterogeneous and structurally complex habitats such as hydrologically dynamic river systems^20,22^. Structurally complex habitats provide predation-refuges for prey species, thus weakening the potential for strong predatory and cascading effects to develop^62^. In our study, the stream bed consisted of a visually complex mix of cobbles, pebbles, gravel and silty detritus, whereas the lake bed was mostly sand and mud with patches of structurally simple macrophytes: quillworts. Although we had assumed the lake bed to be more homogeneous than the stream bed, the quillworts may have introduced structural complexity that prevented effective foraging by platypuses and provided refuges for their prey. Platypuses readily turn rocks and sift through coarse gravelly substrata with their bills, but the bill morphology and foraging mode may be unsuited to foraging through macrophyte beds. The frequent nesting of invertebrates within treatment replicates in the lake provides some evidence that these simple yet spiky plants provide patchy refuges for invertebrates from foraging platypuses. Macrophytes provide refuges from fish predation for many invertebrate prey species^63^, and the morphological and behavioural traits of different fish species show variation in impact on prey residing in macrophytes^64^. Studies comparing the differences in foraging modes and niche overlap of platypuses and fish may help to unravel the roles these very different vertebrates play in freshwater food webs where they co-occur. Alternatively, it is possible that the lake bed itself was more structurally complex than we had appreciated, with invertebrates extending deep into the sediment layers. Deeply buried invertebrates would have been immune to platypus foraging and to our sampling, and could have rapidly replenished prey taken from the surface layers. We cannot reject this possibility, but consider it unlikely owing to the firm and ostensibly unlayered substrates that we used to secure our treatment cages.

Although we had predicted platypuses to indirectly facilitate increases in the biomass of epilithic algae, the absence of strong predatory effects on the herbivore trophic group effectively truncated the cascade. If herbivores found refuges in aquatic macrophytes, then it is possible that these plants provided an indirect commensal benefit to algae within the same trophic level^14^. Experimental removal of macrophytes would help to untangle these possibilities in future work. The impact of platypuses on invertebrate abundance in the stream experiment nonetheless suggests high turnover in the invertebrate assemblage, and indicates that platypuses, like fish in some systems^65^, are likely to be important in nutrient cycling. The absence of platypus-impacts in the lake system clearly indicates that impacts of the same predator may be ecosystem-dependent. We suggest that further studies be carried out in more streams and lake systems, and in different seasons, to confirm the generality of our results.

## Methods

### Study areas

The lotic exclosure experiment was conducted in Brogers Creek, a westerly flowing stream arising near the town of Nowra on the south coast of New South Wales, Australia (34°44′ S, 150°35′ E), an area with warm summers and cool winters. The stream winds through a steep valley surrounded by dairy farms, with riparian vegetation consisting of an undisturbed overstorey of river oaks (*Casuarina cunninghamiana*) with an understorey of sedges (*Lomandra longifolia*), introduced grasses and herbs. River oaks are the main source of litter input, dropping needle-like cladodes and small branches. The dairy farms contribute some organic matter as run-off, although no eutrophication was observed. The stream depth is 2 m maximum, but usually ≤ 1–1.5 m. The substratum is a mix of boulders, gravel, pebbles and cobbles, with silt and detritus in slow-flowing backwaters. A large population of platypuses was resident in the stream during the study, with over 78 individuals captured from August 1998 to August 2001, and individuals travelled its length while foraging^44^.

The lentic exclosure experiment was conducted in Lake Lea, a small (142 ha), shallow, relatively undisturbed sub-alpine dystrophic lake in north-western Tasmania (41°30′S, 146°5′E)^45^. Water depth is mostly 1–2 m, with one hole over 10 m deep. The lake substrate is mostly mud and sand, with some large areas of stone and rocky outcrops. Despite being relatively thinly vegetated, diverse macrophytes are present^45^ with extensive but patchy beds of submerged quillwort (*Isoetes drumondii*) that provide structural habitat and food for aquatic invertebrates. Platypuses and introduced brown trout (*Salmo trutta*) are the main vertebrate predators of invertebrates in the lake. We selected this lake as natural undisturbed freshwater lakes are rare in mainland Australia, and none have been studied with respect to platypus. Lake Lea, by contrast, has a large and well-studied population of platypuses^32–34,46–48^. Prior to our experiment, 52 individual platypuses were captured^48^. However, as Bethge^48^ did not sample the entire lake, the population probably exceeded 52 animals. The exclosure experiment was conducted in the north west of the lake, to avoid interference by anglers^48^.

### Experimental design

Because of the paucity of exclosure experiments investigating the impacts of aquatic mammals on their prey, we briefly reviewed equivalent studies on terrestrial mammals to seek information on appropriate exclosure size, replication and design. Experiments excluding insectivorous mammals, although scant, have used sheet metal or nylon mesh as barrier materials, creating exclusion plots of 3 × 3 m^40,41^. These experiments used 3–4 exclusion plots and 3–4 control plots, and reported rapid increases in numbers of spiders^40^ and of large invertebrates^41^. In further experiments, Wise and Chen^42^ excluded all vertebrates from 50 m^2^ plots (*n* = 5 treatments, 5 controls), but detected no effect on densities of wolf spiders. A review of the effects of predator removal on terrestrial vertebrate prey found that 23 of 116 experiments used exclosures^43^. Of these, only 13 studies reported any replication, this ranged from 2 to 4 removal plots and an equal number of control plots in all cases^43^. The median size of plots was 2 ha, reflecting the larger spatial requirements of vertebrate compared to invertebrate prey. Despite the possibility that exclusion fences might affect prey, only two studies reported the use of procedural controls (i.e. sham fences)^40,66^, all others used open control plots to compare the effects of predator exclusion^43^.

Following this review, we ran an exclosure experiment in the stream from late summer through autumn 1999 and in the lake from late summer to autumn 2000. The experiments were designed to examine the impact of platypuses on the abundance, taxon richness and community structure of benthic invertebrates, as well as on sediment and epilithic algal biomass. We used three treatments in each of these two contrasting experimental systems: exclosure cages (− PLATYPUS) which prevented access by platypuses to the substrata; uncaged benthic areas (+ PLATYPUS) where platypuses had free access; and a procedural control to determine any cage effects (+ PLATYPUS control).

In the stream, we selected a large pool, ~ 100 m in length, bounded upstream and downstream by 10–20 m long riffles, and installed four mesh cages to exclude platypuses (− PLATYPUS treatment). As noted above, this level of replication is similar to, or greater than, that in most terrestrial exclosure experiments. All cages (1.2 m × 1.2 m, 30 cm high) were constructed of brown plastic Nylex^®^ garden mesh (mesh dimension 5 × 5 cm). Five extra holes, 5 × 10 cm, vertically aligned, were cut in the mesh on all sides and at the top of the cages. These holes, and mesh size, while excluding platypuses, allowed access by invertebrates and fish, including adults of larger fish in the system—Australian bass (*Macquaria novemaculeata),* long-finned eels (*Anguilla reinhardtii*), and short-finned eels (*Anguilla australis*). As judged by the free movement of leaf litter and detritus in water through the cages, the cages had minimal or no effect on water current velocity. Four additional mesh cages of the same dimensions were installed as procedural controls (+ PLATYPUS control) but had 25 × 25 cm holes in the sides and top. These cages allowed free access by platypuses yet still approximated any influence of the cage structure on movements of platypuses, fish, and invertebrates. Plastic mesh was used to prevent any possible interference with platypus electroreception during feeding^49^. In addition to the mesh cages, four open, uncaged plots the same dimensions as the cages were marked on the open stream bed to serve as open treatments (+ PLATYPUS).

The cage mesh was secured to the substrate using metal stakes and rocks. To simulate this disturbance for all treatments, including the open treatment, rocks were similarly displaced. Cages were placed at the downstream end of the pool where current velocity was minimal, at least 2 m from the stream edges to avoid any systematic differences in current velocity due to the stream banks. Although treatments were confined to broadly the same area, and thus were exposed to similar environmental conditions, we stratified the placement of cages in water depths of 0.45–1.25 m to ensure more representative sampling of the environment. We also placed cages with opposite corners in line with stream flow to minimise leaf litter accumulation on the upstream edge. Treatment plots were ≥ 3 m apart; as the benthic prey of platypuses was expected to be largely sessile, this separation was considered sufficient to avoid spatial confounding. Within these constraints, cages were set in random locations, with assignment to treatment made at random. A single post driven into the substrate was used to mark locations of the + PLATYPUS treatment replicates.

Within each treatment replicate a sediment trap consisting of a plastic tube 10 cm high, with a 4.5 cm diameter opening, was fixed vertically to a stake ~ 20 cm inside the downstream corner of the cage. Sediment traps were used to collect benthic sediments disturbed and suspended by platypus foraging activities or other disturbances. Also, a pre-conditioned terracotta tile (20 cm^2^) was placed in the middle of each cage, or in the case of the + PLATYPUS treatment, about 20 cm upstream of the sediment tube/marker post to determine if platypuses had any direct or indirect effects on epilithic algae. If platypuses suppress algal-grazing herbivorous invertebrates, it is likely that algal abundance would vary differentially between treatments on the artificial tile substrates. Tiles were preconditioned by leaching them in the river for six weeks prior to the experiment, and any accumulated algae were removed before deployment.

The exclosure experiment in the lake was similar to that conducted in the stream, except that six replicates of each treatment were used rather than four. This increased statistical power to detect any treatment differences, given that the lake was expected to have lower invertebrate biomass compared with the stream. Treatment plots were again ≥ 3 m apart, set up on sites where the substrate was firm enough to support the cages, and treatments allocated randomly. Platypuses are larger in Tasmania than on mainland Australia, but still much smaller than the holes in the procedural control cages and thus able to readily pass through them. Brown trout (*Salmo trutta*) in the lake are 0.6–1 kg, but rarely reach this size (https://www.ifs.tas.gov.au/ifs/IFSDatabaseManager/WatersDatabase/lake-lea), so individuals could readily pass through all the exclosures.

Both experiments ran for six weeks before invertebrate sampling took place. Six weeks was deemed long enough for any potential effects of platypus foraging to be detected, especially as the late summer to autumn study period is when male platypuses attain their greatest body mass and condition and could be expected to forage most intensively^29,44^. Conversely, a more prolonged experimental period would have seen increasing damage to the exclosure structures from both water flow and human interference. We did not repeat the experiments in winter through spring to avoid disturbance to the platypus breeding season^44^. However, there is little or no seasonal variation in the composition of aquatic invertebrates between seasons, at least in the stream system^29^. This may suggest that similar results could be obtained at other times, although further experiments are needed to confirm this. At least 14 platypuses were known to have moved through the experimental stream pool over the study period, with some individuals visiting the open and control treatments, based on capture and radiotracking data^44^. In comparison, only five Australian bass were captured during extensive net sampling during the same period, suggesting that, during the course of the experiment, platypus abundance exceeded that of the most abundant large predatory fish in the pool^44^. Platypuses were probably present in much greater numbers in the pool than those identified, as platypuses in this system have large and overlapping linear home ranges^44^, and numbers were not monitored continuously during the experiment.

Ideally the experiments would have been replicated in multiple streams and lakes to increase the power and generality of our results, and to have been run across different seasons, but this was not logistically possible. We therefore interpret our results with caution and note that our conclusions are restricted to the sites and seasons that were studied.

### Invertebrate, algal, and sediment sampling

Invertebrates were sampled by day in both systems using a Brooks suction sampler (Brooks^67^ (33 cm^2^ sampling area). Although 33 cm^2^ is relatively small, pilot studies suggested that this area would yield sufficient invertebrates to allow robust tests of our hypotheses. However, because we also expected small-scale spatial variation in the invertebrates, we took three sub-samples of invertebrates in each replicate cage. Suctioning for each sample took 60 s, with the sampler held firmly over the substrate. Samples were then preserved separately in 70% ethanol and transported to the laboratory for identification.

Invertebrates were sorted from the detritus under × 6–× 40 magnification, counted, and identified to genus where possible^68^. Exceptions, due to taxonomic impediments, were fly larvae of the families Chironomidae and Tipulidae, aquatic mites (Acarina), worms (Oligochaeta), flatworms (Dugesiidae), and members of the beetle family Scirtidae. Invertebrates were assigned to a trophic group (detritivore, herbivore, omnivore, predator) using published accounts^36,50^ and following our previous work^29,44^. These assignations are approximate as diets can vary between instars and locales. However, the categories were considered to be broadly useful in determining functional roles^51^ and thus for elucidating the role of platypuses in predator–prey and potentially trophic cascades in the study ecosystems. Leaf litter detritus from the stream samples was retained, dried and weighed, but these data are not presented as allochthonous leaf litter was not common in the lake, thus preventing direct comparisons^44^.

At the conclusion of both experiments, six weeks after exclosure establishment, algae were vigorously brushed from the tiles, washed into vials using stream or lake water and preserved using 2% Lugol’s iodine solution. In the laboratory, algae were filtered onto pre-weighed 0.45 μm filterpaper, dried at 60 °C to constant weight, and weighed to 0.0001 g. Sediment traps were collected and the material was transferred to a pre-weighed drying dish and dried to constant weight at 60 °C. The material was then weighed to 0.01 g precision.

### Data analysis

All analyses focused on comparisons between the three treatments (i.e., + PLATYPUS, − PLATYPUS and + PLATYPUS control) within each experiment, but separately between the lotic and lentic systems. Two sets of analyses were undertaken for the two ecosystem datasets. Firstly, univariate comparisons were carried out to identify differences among means for the abundance and taxon richness of invertebrates and invertebrate trophic groups (hypotheses 1 and 2), algal biomass and sediment mass (hypotheses 3 and 4). Secondly, multivariate analyses were carried out to explore possible shifts in composition of the invertebrate community as a whole among treatments, separately in both systems (hypothesis 2). We did not formally compare the datasets observed in the lentic and lotic systems (hypothesis 5), but instead compared the effect sizes arising from the manipulation of platypus in each system.

Univariate data were subjected to Cochran’s test for homogeneity of variances^52^. Due to heterogeneity, invertebrate data were √-transformed, then analysed using a nested (hierarchical) one-factor analysis of variance (ANOVA), with treatments fixed, and replicates nested within treatments^52^. We took this approach to quantify replicate-within-treatment variance, rather than losing information by averaging across samples^52^. Algae and sediment data were also √-transformed and compared among treatments using a one-factor ANOVA. Tukey’s multiple comparison test was performed on each pair-wise comparison to identify sources of difference between treatments. Univariate tests were conducted using SYSTAT version 9 and Statistica 13.

The multivariate invertebrate community dataset was analysed using PRIMER, version 5. Community composition within each treatment was first assessed using a Bray–Curtis dissimilarity matrix. This distance measure is widely used in ecological studies, and is considered to be robust^53,54^ and useful in determining the underlying structure of biological communities. The matrix was then subjected to non-metric multidimensional scaling (nMDS), providing an ordination where the distance between samples reflects relative similarity in species composition. Data were square root transformed to down-weight the effects of the most common taxa and maintains the effects of the less common taxa^29,55^. An analysis of similarity (ANOSIM routine, PRIMER ver. 5) was performed on the dissimilarity matrices to test for differences between treatments. This permutation test uses a randomisation approach to generate significance levels to test a priori hypotheses about differences between groups of samples^54,55^. The SIMPER (Similarity Percentages) sub-routine in Primer ver. 5^55^ was used to examine the contribution of each taxon to the average dissimilarity between all pairs of inter-group samples. This test does not have a statistical hypothesis-testing framework, but is useful in data exploration to indicate which ‘taxa’ are principally responsible for differences between a priori defined groups that differ in matrix structure^55^. SIMPER was used to determine which trophic groups contributed to dissimilarities between the + PLATYPUS and − PLATYPUS treatments.

## Data Availability

The datasets generated during and analysed during this study are available from the corresponding author on reasonable request.
